# Dialectics of Imagination and Experimentation: Basic Science Research in Developing Countries

**DOI:** 10.12669/pjms.331.12591

**Published:** 2017

**Authors:** HR Ahmad, NA Khan

**Affiliations:** 1 HR Ahmad, Department of Biological and Biomedical Sciences Aga Khan University, Karachi Pakistan. E-mail: hrahmad.alrazi@aku.edu; 2 NA Khan. Department of Biological Sciences, Sunway University Malaysia

The cycle of imagination and experimentation is the key for any research discoveries or pathways. The ability to imagine (hypothesis) and then test it through experimentation would yield a scientific conclusion. This process is known as a hypothesis-based research. Conversely, in a non-hypothesis-based research pathway, experimentation leads to data analysis that can be explained with the help of a hypothesis. This leads to a scientific theory that can in turn become a law upon repeated observations by a wider scientific community. These two pathways are alternatively used in research. However, later should be preferred for it enters into the axis of theory and law. Since imagination and experimentation are vital elements for research discovery, they are considered as the benchmark traits of a scientist. The combination of both elements is the pre-requisite to become a genuine scientist. For great artists like Picasso, hands are the ambassadors of the brain and vice versa. Similarly, both imagination (brain) and experimentation (hands) must be displayed to be a creative scientist. For a developing country like Pakistan research is mostly decoupled between imagination and experimentation. The creative energy and the spirit of inquiry (imagination) can be unfolded by laboratory work (experimentation) and the horizontal enlightened enabling research environment. Given the deficiencies in research infrastructure with limited technical expertise in the third world settings, there is a need to do more thinking. The study of basic science disciplines (anatomy, biochemistry, cell and molecular biology, physiology and pharmacology) of Homo sapiens can be pursued despite aforementioned challenges. What we fail to understand is that Homo sapiens are one of the millions of species in the kingdom. All of these basic science disciplines can be very well studied in a cost/time effective manner with ethical adherence to other species of the Kingdom. Since many of the species have a life span from few minutes to few hours with conserved molecular pathways, do not require ethical approval, millions are available for experimentation with meaningful ‘n’ values should invite scientists to imagine and test their ideas through experimentation. For example, much of what is known today of motility (actin and myosin) comes from our knowledge gained from our ancestral amoeba, much of the knowledge of biochemistry comes from microbes, C. elegans has provided us with the mechanism of apoptosis and the lice brain has provided us with mechanisms of potassium homeostasis. Many of these species can be experimented to research anatomy (e.g. skeletal system of microbes), biochemistry (cell and molecular biology and genetics), physiology (life function of microbes), pathology and pharmacology (effects of compounds on cells, molecules and genes). In addition, these species can be used to study complex behaviors in a given population and effects of environment. As behavior is determined by interactions of both genes and environment, this can be studied much more easily in microbes to be compared with Homo sapiens.

